# If Digital Literacy is an Overarching Determinant of Health, how can we measure and confirm e-literacy in older adults?

**DOI:** 10.1093/geroni/igaf122.3792

**Published:** 2025-12-31

**Authors:** Michael Splaine, Nora Reder, Kate Gordon

**Affiliations:** Splaine Consulting, Columbia, Maryland, United States; University of California, Berkeley, Berkeley, California, United States; Splaine Consulting, Columbia, Maryland, United States

## Abstract

Older adults need to navigate an increasingly digital world-and the heterogeneity of the population’s digital skills (and those of their family caregivers) can be a facilitator or a barrier to care and support and best lives. This poster presents the case for viewing digital literacy as a social determinant of health and a summary of the research validated measures of digital literacy that can be put into place in health and social care organizations. The last portion of the paper outlines a process which can be used to adopt in measures and to assist older adults with access to digital competence and tools.

